# Transcription analysis in the MeLiM swine model identifies RACK1 as a potential marker of malignancy for human melanocytic proliferation

**DOI:** 10.1186/1476-4598-7-34

**Published:** 2008-04-28

**Authors:** Giorgia Egidy, Sophia Julé, Philippe Bossé, Florence Bernex, Claudine Geffrotin, Silvia Vincent-Naulleau, Vratislav Horak, Xavier Sastre-Garau, Jean-Jacques Panthier

**Affiliations:** 1 INRA, UMR955 Génétique Moléculaire et Cellulaire; Laboratoire conventionné CEA n°17 ; Ecole Nationale Vétérinaire d'Alfort, 7 avenue du Général de Gaulle, Maisons-Alfort, F-94704 France; 2 Institut Pasteur, Unité de Génétique Fonctionnelle de la Souris; CNRS URA 2578, Département de Biologie du Développement, USC INRA, 25 rue du Dr. Roux, Paris, F-75724 France; 3 Ecole Nationale Vétérinaire d'Alfort, Service d'Anatomie pathologique, 7 avenue de Général de Gaulle, Maisons-Alfort, F-94704 France; 4 CEA, DSV, IRCM, SREIT, Laboratoire de Radiobiologie et d'Etude du Génome, INRA Jouy-en-Josas, F-78352 France; 5 INRA, DGA, Laboratoire de Radiobiologie et d'Etude du Génome, Jouy-en-Josas, F-78352 France; 6 Institute of Animal Physiology and Genetics, 27721 Libechov, Czech Republic; 7 Institut Curie, Service d'Anatomopathologie, 26 rue d'Ulm, Paris, F-75005 France

## Abstract

**Background:**

Metastatic melanoma is a severe disease. Few experimental animal models of metastatic melanoma exist. MeLiM minipigs exhibit spontaneous melanoma. Cutaneous and metastatic lesions are histologically similar to human's. However, most of them eventually spontaneously regress. Our purpose was to investigate whether the MeLiM model could reveal markers of malignancy in human melanocytic proliferations.

**Results:**

We compared the serial analysis of gene expression (SAGE) between normal pig skin melanocytes and melanoma cells from an early pulmonary metastasis of MeLiM minipigs. Tag identification revealed 55 regulated genes, including *GNB2L1 *which was found upregulated in the melanoma library. *In situ *hybridisation confirmed *GNB2L1 *overexpression in MeLiM melanocytic lesions. *GNB2L1 *encodes the adaptor protein RACK1, recently shown to influence melanoma cell lines tumorigenicity. We studied the expression of RACK1 by immunofluorescence and confocal microscopy in tissues specimens of normal skin, in cutaneous and metastatic melanoma developped in MeLiM minipigs and in human patients. In pig and human samples, the results were similar. RACK1 protein was not detected in normal epidermal melanocytes. By contrast, RACK1 signal was highly increased in the cytoplasm of all melanocytic cells of superficial spreading melanoma, recurrent dermal lesions and metastatic melanoma. RACK1 partially colocalised with activated PKCαβ. In pig metastases, additional nuclear RACK1 did not associate to BDNF expression. In human nevi, the RACK1 signal was low.

**Conclusion:**

RACK1 overexpression detected *in situ *in human melanoma specimens characterized cutaneous and metastatic melanoma raising the possibility that RACK1 can be a potential marker of malignancy in human melanoma. The MeLiM strain provides a relevant model for exploring mechanisms of melanocytic malignant transformation in humans. This study may contribute to a better understanding of melanoma pathophysiology and to progress in diagnosis.

## Background

Cutaneous melanoma is a malignant tumor developing by transformation of melanocytes. Its worldwide incidence and mortality rate in fair-skinned populations are on the increase. Presence of metastases carries a severe prognosis because efficacious systemic treatments are still lacking. An earlier detection of the primary melanoma would help improve prognosis. To this aim, markers identifying malignant lesions are needed. Moreover, understanding the molecular bases of oncogenicity in melanocytic proliferation may contribute to the development of efficacious therapies. Among the animal models, the MeLiM (Melanoblastoma-bearing Libechov Minipig) strain affected by cutaneous melanoma is of particular interest. This swine model has been recently characterized [1-3]. Familial predisposition to cutaneous melanoma in MeLiM is neither linked to the *CDKN2A *gene [4] nor to *BRAF *[2], but rather depends on the complex interactions between multiple genes [3]. In MeLiM, cutaneous tumors develop *in utero *or in the first 3 months after birth with an incidence reaching 50%. The cutaneous tumors disseminate to inner organs, with the highest incidence in lymph nodes. However, MeLiM tumors and human melanomas show a major difference in outcome: MeLiM melanomas present a high propensity to regress, by contrast with human melanomas [1,5].

To determine whether the MeLiM model could provide valuable information on markers of the disease in humans, we decided to identify genes involved in melanocytic proliferation in MeLiM and to then assess their expression in human specimens of normal skin as well as benign and malignant melanocytic lesions. The serial analysis of gene expression (SAGE) technology was chosen because, unlike microarrays, it gives a complete profile of the gene expression in the cells, regardless of the sequences to be analysed. SAGE libraries can be compared *in silico *to reveal genes specifically expressed in certain cell types [6]. Interfollicular melanocytes make up 4% of the cells in normal epidermis. To minimise the contribution of cells other than melanocytes, we constructed SAGE libraries from PigMel melanocytes derived from the skin of a healthy Meishan minipig [7] and from primary melanoma cells cultured from pulmonary melanoma metastases in MeLiM. We report here the differences in gene expression between malignant and normal melanocytes. The pattern of expression detected *in situ *in pig specimens of one of these genes, encoding RACK1, was confirmed in human melanocytic lesions. Our results unveil a marker of malignancy for human melanocytic proliferation.

## Results

### Comparative expression analysis between pig metastatic melanoma cells and melanocytes

Young MeLiM developed melanoma metastases in lymph nodes, liver, heart and lung. To isolate melanoma cells from lung metastases, primary cultures of tumors were performed under conditions optimised for pig melanocyte proliferation [7]. After 48 hr in culture, adherent cells were predominantly melanocytes. SAGE libraries were constructed from 2.5 millions of these metastatic melanoma cells (MMC) and control PigMel normal melanocytes (NM). A total of 11,700 and 11,300 tags were sequenced from the MMC and NM libraries, corresponding to 6,131 and 5,466 different tags (transcripts), respectively. Our data have been deposited at NCBI's Gene Expression Omnibus [8] and are accessible through the GEO Series number GSE5982.

To identify genes potentially involved in malignant progression, we compared the two libraries. Monte Carlo simulations yielded 70 tags statistically significant at *p *value < 0.05. Fifty-five (79%) matched expressed sequence tags (EST), the remaining 15 (21%) tags presented no matches. A majority of tags matched genes expressed at high levels. Among the EST, 39 (56% of tags) matched to known cDNAs, the remaining 16 (23%) could not be identified. The identified genes are involved in RNA processing and protein synthesis (20% of the 70 tags), transcription (7%), signalling (4%) and the rest corresponded to scattered functional classes (24%). The list of tags increased and decreased in MMC compared to NM, arbitrarily ordered by functional classes of the genes they represent, are shown in Tables 1 and 2, respectively. Several of the genes in Table 1 have been shown to be differentially expressed in various tumors, compared to their normal counterparts. *RPS12*, *Secernin*, *CDC10 *were found to be upregulated in human colorectal tumors, gastric cancers, diffuse large B-cell lymphomas, respectively [9-11]. Similarly, genes listed in Table 2 like *COXIII*, were found to be downregulated in human glioblastoma [12]. The mRNA of *GNB2L1 *corresponded to a tag abundant 31 and 13 counts in MMC and NM, respectively. We chose to study *GNB2L1 *expression in melanocytes and melanomas in more detail because *GNB2L1 *encodes RACK1, receptor for activated C kinase, whose mRNA was found to be up-regulated in human carcinomas [13].

**Table 1:** SAGE tags significantly increased in metastatic melanoma cells (MMC) compared to normal melanocytes (NM)

GATC preceded **Tag**	**Count**		**p value**	**GenBank match [accession number]**
			
	**NM**	**MMC**		
**Translation, ribosomal structure and biogenesis**				

ACATCCATCA	28	73	0	Ss 40S ribosomal protein S20, RPS20 [AY550070]
AAACCAAAGA	1	10	0.004	Ss cDNA [AJ681350]homolog to Hs small nuclear ribo- nucleoprotein polypeptides B and B1, SNRPB [BC080516]
TGACTATAAC	18	35	0.007	Ss cDNA [BX923125] homolog to Hs ribosomal protein L24, RPL24 [BC000690]
AAGTTCCCGC	17	32	0.012	Ss cDNA [BX674755] homolog to Hs ribosomal protein L18a, RPL18A [BC066319]
AACCTAATTA	58	77	0.025	Ss 40S ribosomal protein S12, RPS12 [NM_214363]
AACTCAATAA	44	62	0.027	Ss ribosomal protein L10a, RPL10A [NM_001097477]
AAAGATTAAG	20	32	0.040	Ss ribosomal protein L27, RPL27 [NM_001097479]
**Signal transduction mechanisms**				

ATTGTAGATG	13	31	0.002	Ss guanine nucleotide beta like protein GNB2L1, RACK1 [NM_214332]
AGTTATGAAG	0	5	0.028	Ss cDNA [CN160727] homolog to Hs ras-related GTP-binding protein, Rheb [D78132]
AAGCTACACA	4	11	0.043	Ss cDNA [CJ016486] homolog to Hs calmodulin 1 [phosphorylase kinase, delta], CALM1 [BC011834]
**Transcription**				

CAGAGGGACA	1	10	0.004	Ss cDNA [CN 157150] homolog to Hs retinoblastoma binding protein, RbAp48 [X74262]
ACAACTGGGG	0	6	0.014	Ss cDNA [CJ020293] homolog to Hs general transcription factor IIB, GTF2B [NM_001514]
TGATAGAAGA	6	15	0.025	Ss cDNA [CF792678] homolog to Hs activating transcription factor 4, ATF4 [BC073754]
TATGAATAAG	4	11	0.043	Ss non-metastatic cells 1 protein NM23A [NM_001044610]
**Secondary metabolites biosynthesis, transport and catabolism**				

AGTATCAACA	24	46	0.002	Ss TYRP1 tyrosinase related protein1 [AB207240]
**Secretion**				

TGCTCAGGCT	0	13	0.000	Ss cDNA [BX674961] homolog to secernin
**Energy production and conversion**				

AATACAAGTT	3	12	0.014	Ss NADH dehydrogenase [ubiquinone]1 alpha subcomplex 4 NDUFA4 [NM_001097468]
**Posttranslational modification, protein turnover**				

GAGGTGGAGA	3	10	0.043	Ss cDNA [CJ014518] homolog to Hs peptidylprolyl isomerase B [cyclophilin B], PPIB [NM_000942]
**General prediction only**				

ATTTCTAGGC	0	5	0.028	Ss cDNA [CK455473] homolog to Hs CDC10 cell division cycle 10 homolog [S. cerevisiae], CDC10 [NM_001788]
**Function unknown**				

TCACCCGCAA	0	9	0.0015	No reliable matches
TCGTCCCTGT	0	8	0.0031	Ss cDNA [BX665592]
CCTGTGCTGA	0	7	0.0064	Ss cDNA [AJ647874]
GGTCATTCAT	0	7	0.0064	No reliable matches
TCGCCTGGAC	0	7	0.0064	No reliable matches
TCGTCCCTTC	0	7	0.0064	No reliable matches
ATTCATGTCA	3	13	0.0079	Ss cDNA [BP164587]
GTCTAATCAC	2	10	0.0134	No reliable matches
AATGACCGAC	0	6	0.0147	No reliable matches
CACCCGCAAT	0	6	0.0149	No reliable matches
CCTTCCGACT	0	6	0.0149	No reliable matches
CGTCCCTGTG	0	6	0.0149	No reliable matches
AGATAATTTG	0	5	0.0285	Ss cDNA clone [EW659942]
ATAGACGAGC	0	5	0.0285	No reliable matches
CTGCATTGCT	1	7	0.0321	Ss cDNA [BX922537]
AGAATATAAG	5	13	0.0341	No reliable matches
CCGCGTTGCT	41	57	0.0362	Ss cDNA [AJ666089]
ATGAAGATAT	2	8	0.0434	Ss cDNA [CB286104]
TGCTGCAGGG	4	11	0.0435	Ss cDNA [CA780804]

**Table 2:** SAGE tags significantly decreased in metastatic melanoma cells (MMC) compared to normal melanocytes (NM)

GATC preceded **Tag**	**Count**		**p value**	**GenBank match [accession number]**
		
	**NM**	**MMC**		
**Translation, ribosomal structure and biogenesis**				

GTCGTTCTGG	52	29	0.015	Ss eukaryotic translation elongation factor 1 alpha 1 [NM_001097418]
GACTTTGACA	6	0	0.016	Ss cDNA [BX676185] homolog to Hs eukaryotic translation initiation factor 3 subunit k, eIF3k [AY245432]
TGTCAAAAAA	22	9	0.025	Ss ribosomal protein L29, RPL29 [NM_213950]
TCTGGAAAGA	24	11	0.027	Ss cDNA [BX675247] homolog to Hs ribosomal protein S2, RPS2 [BC019021]
TCTGACTACC	5	0	0.031	Ss cDNA [BX922533] homolog to Hs mitochondrial ribosomal protein L40, MRPL40 [NM_003776]
AGAAAGCTGT	9	2	0.042	Ss ribosomal protein L6, RPL6 [NM_001044542]
AGCGTTCAGC	39	23	0.049	Ss 40S ribosomal protein S16, RPS16 [AY550068]
**Transcription**				

AGGGGAAATG	11	3	0.041	Ss cDNA [BX665090] homolog to Hs small nuclear ribonucleoprotein D3, [BC034447]
**Secondary metabolites biosynthesis**				

ACCCTGGCTG	90	46	0.000	Ss cDNA [BX 920958] homolog to Equus caballus melanocyte protein 17 precursor, PMEL17 [AF076780]
CACTGCTCAA	16	6	0.038	Ss glycoprotein [transmembrane] nmb GPNMB [NM_001098584]
**Energy production and conversion**				

CTAAAAAAAA	18	5	0.006	Ss mitochondrial COX III [AJ953126]
TCAGAAGAGA	15	4	0.012	Ss cDNA [CN155299] homolog to Hs ATP synthase, H+ transporting, mitochondrial F1 complex, a subunit, isoform 1, ATP5A1 [BC008028]
TCACCTGGGG	9	2	0.042	Ss cDNA [CB477260] homolog to Hs ATP synthase, H+ transporting, mitochondrial F1 complex, epsilon subunit, ATPE [BC003671]
**Cell division**				

CCCAACAATG	5	0	0.031	Ss beta 5-tubulin, [NM_001044612]
**Posttranslational modification, protein turnover**				

TCTAAAGCGG	7	0	0.009	Ss cDNA [BX672659] homolog to Hs glucose regulated protein 58 KD, GRP58 [NM_005313]
AGTGGCTTTG	7	1	0.043	Ss cDNA [CJ007805] homolog to Hs proteasome [prosome, macropain] subunit, beta type, 4, HsN3 [BC008314]
AGGGAATGGA	5	0	0.031	Ss cathepsin B, CTSB [NM_001097458]
**General prediction only**				

AGCATCCAGA	5	0	0.031	Ss cDNA [BX925627] homolog to Hs nuclear distribution gene C homolog, NUDC [NM_006600]
CAGAGCTGCC	5	0	0.031	Ss cDNA [CK459123] homolog to Hs SET binding factor 1, SBF1 [NM_002972]
CTAGACGACT	7	1	0.043	Ss cDNA [BX676609] homolog to Hs arginine-rich, mutated in early stage tumors, ARMET [NM_006010]
**Function unknown**				

AGATGGCCAG	7	0	0.009	No reliable matches
AGCTTAAGCA	6	0	0.016	Ss cDNA [BX922388]
ATGTGCCTGG	6	0	0.016	Ss cDNA [BX674457]
AGACTTTTAA	6	0	0.016	Ss cDNA [EW337487]
TATGGGGGTC	6	0	0.016	Ss cDNA [EW673617]
AGCCTGGACC	5	0	0.031	No reliable matches
CCTAGCCTGG	5	0	0.031	Ss cDNA [DY430777]
TGGCATGGCT	5	0	0.031	Ss cDNA [BX920198]
AGCTGTTCTA	11	3	0.041	Ss cDNA [BX670542]
CACCCGCAAT	9	2	0.042	No reliable matches
AGTCCCTGTG	7	1	0.043	No reliable matches
TTTGCAAGGG	7	1	0.043	Ss cDNA [CJ006347]

### *RACK1 *mRNA overexpression in MeLiM melanoma

**Figure 1:**
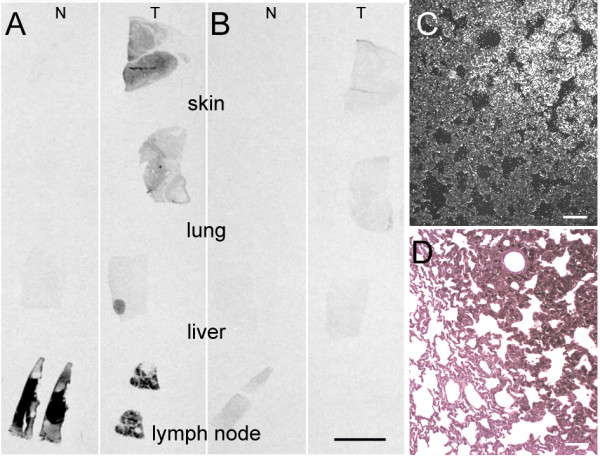
**Expression of *RACK1 *mRNA in pig tissues**. (A-B) *In situ *hybridisation autoradiography of *RACK1 *antisense (A) and sense (B) probes on depigmented sections. For each probe, normal (N) tissues are displayed on the left (skin, lung, liver and lymph node) and tumoral (T) tissues on the right (cutaneous melanoma, metastatic melanoma from lung, liver and lymph node). Note the intense signal on the tumors compared to the healthy or non-compromised tissues, with the antisense probe, except for lymph node. (C) Darkfield photomicrograph taken from a MeLiM melanoma lung metastasis hybridised with the *RACK1 *antisense probe. (D) Consecutive section stained with hematoxylin and eosin. The pigmented area in the tumor matches the region which exhibits silver grains on (C). Bar = 1 cm for A and B and 100 μm for C and D.

To define the distribution pattern of *RACK1 *mRNA, we performed *in situ *hybridisation onto pig sections of normal skin, and on samples of cutaneous melanoma and metastatic melanoma samples from lung, liver and lymph nodes. To avoid background in heavily melanogenic tumor areas, a bleaching treatment was added. Film autoradiography obtained with the antisense and sense probes showed a faint signal of *RACK1 *mRNA in healthy tissues, except in the lymph nodes where the signal was strong (Figure 1A), as reported in human lymph nodes [13]. By contrast, an intense signal was observed in tumoral regions of cutaneous melanoma, lung and liver metastasis samples; non-tumoral regions displayed a much lower signal (Figure 1A). The sense probe autoradiographic signal was almost negligible (Figure 1B). Darkfield illumination on emulsion autoradiography highlighted the silver grains on the tumoral region of lung melanoma (Figure 1C). These results confirmed the overexpression of *RACK1 *mRNA in melanoma, as predicted by our SAGE data.

### RACK1 protein localisation in skin, primary and metastatic melanoma in pigs

**Figure 2:**
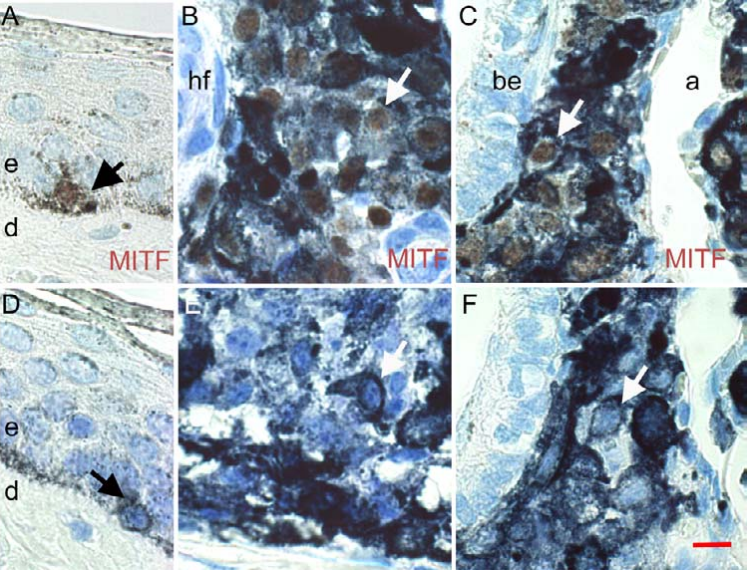
**Identification of melanoma cells from MeLiM by MITF**. Melanocytes were visualized as brown nuclear granules, by immunohistochemistry with MITF antibody (A-C) and without the primary antibody (D-F). (A, D) Normal skin. (B, E) Cutaneous melanoma. (C, F) Melanoma metastasis in a lung. a, alveolae; be, bronchiolar epithelium; d, dermis; e, epidermis; hf, hair follicle. Arrows point to normal melanocytes (black) and melanoma cells (white). Bar = 100 μm.

On tissue sections, identification of melanoma cells was achieved with an antibody against the microphthalmia transcription factor MITF, which produces a specific nuclear signal in melanocytes. Immunohistochemistry showed that normal melanocytes in the basal layer of control skin epidermis, were labelled for MITF (Figure 2A). Tumoral cells in cutaneous melanoma as well as in metastases were also labelled with the MITF antibody (Figure 2B, C). Unspecific labelling was not detected with this antibody (Figure 2D–F). Thus, MITF is a sensitive marker of the melanocytic lineage, useful to study melanoma progression in the pig.

**Figure 3:**
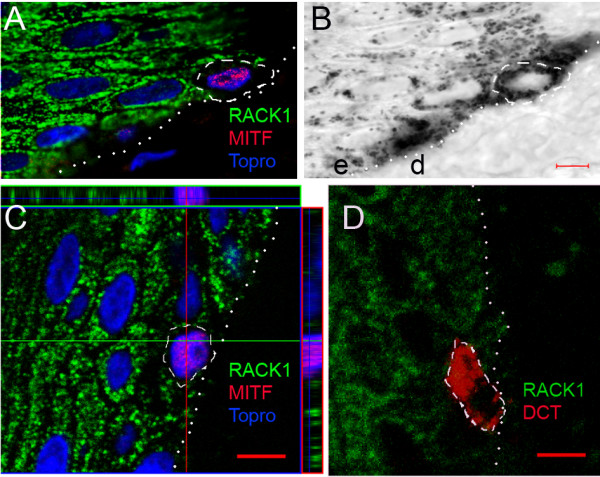
**RACK1 expression in normal pig epidermis**. (A, C, D) Confocal microscopy analysis of RACK1 protein (green fluorescence), and double labelling for either MITF (A, C) or DCT (D) (red fluorescence) on pig skin. Normal epidermis were from control Meishan minipig (A, B), and MeLiM (C, D). (B) Transmission photograph corresponding to (A). (C) Three dimensional 'orthogonal' slice projection analysis is included: the large central panel shows a single optical slice through which an x axis (green line) and a y axis (red line) were defined for sliced z-axis reconstruction. The corresponding results for the x, z slice (top) and the y, z slice (right) are shown. The blue line represents the position of the central panel image in the z stack. Nuclear counterstaining is shown in blue. Note the RACK1 cytosolic spotty signal on keratinocytes and its absence in the melanocyte indicated by the white dashed line. Dotted lines indicate epidermis-dermis boundaries. e, epidermis; d, dermis. Bar = 5 μm.

To explore whether overexpressed *RACK1 *mRNA was translated, we analysed the cellular distribution of RACK1 protein by confocal microscopy, with double immunostaining for MITF. In control Meishan and healthy MeLiM skins, RACK1 protein was expressed in the epidermis, and found in the cytoplasm of keratinocyte (Figure 3A–D). In MITF-positive (MITF+) melanocytes, RACK1 expression was not detected (Figure 3A, C). Consistent with this, when testing dopachrome tautomerase (DCT), a melanogenic enzyme restricted to melanosomes, double labelling of RACK1 and DCT did not overlap in normal skin (Figure 3D).

**Figure 4:**
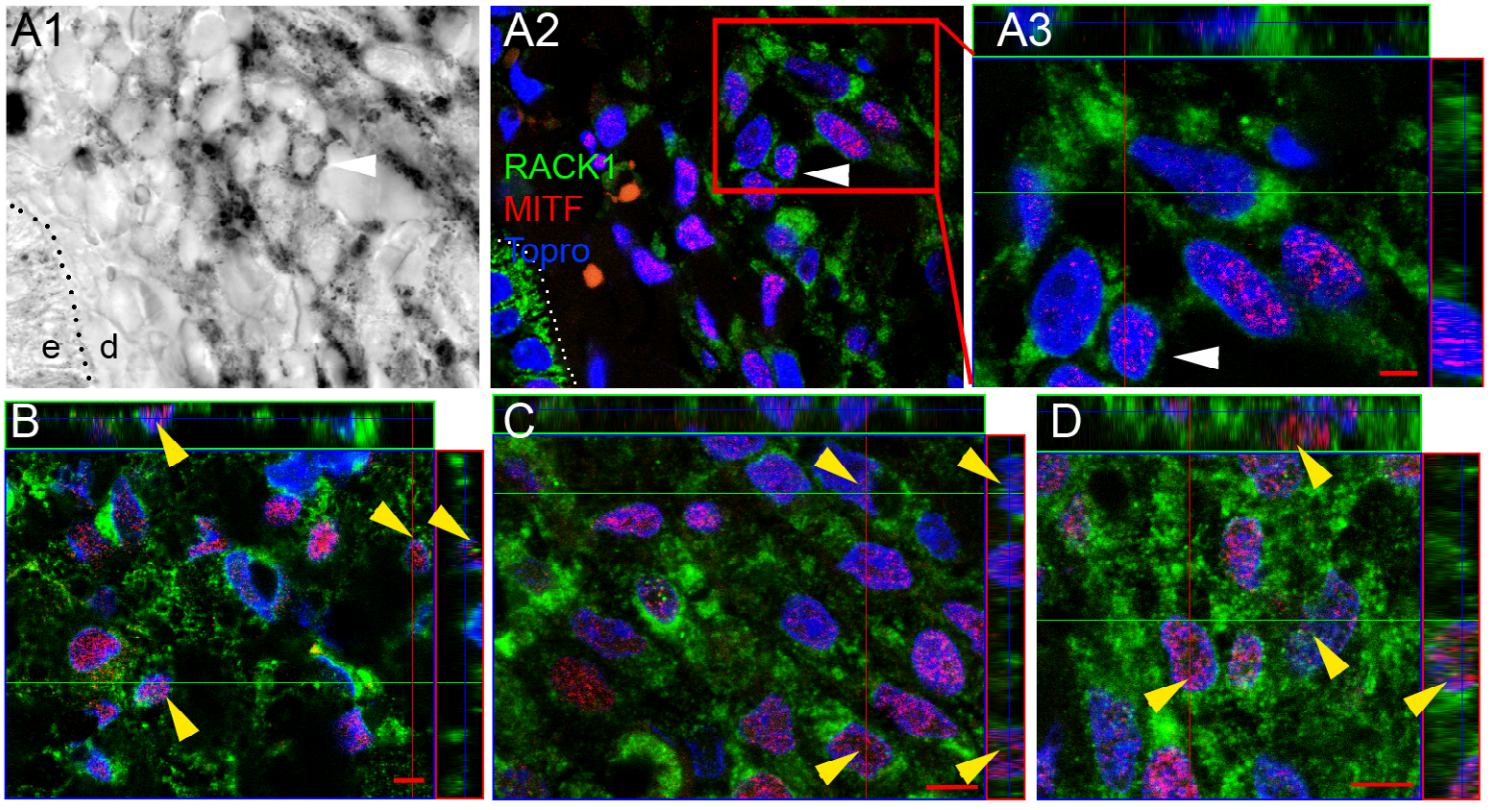
**Cellular distribution of RACK1 in MeLiM melanoma at different progression stages**. Confocal microscopy analysis of RACK1 (green fluorescence), and double labelling for MITF (red fluorescence). (A) Cutaneous melanoma. (B-D) Melanoma metastasis in a lymph node (B), lung (C) and heart (D). Three dimensional 'orthogonal' slice projection analyses are presented as in Figure 3. Nuclei are shown in blue. (A1) Transmission photograph corresponding to (A2). (A3) Zoom on (A2) inset. White arrowheads in (A1-A3) point at a dermal cutaneous melanoma cell positive for MITF and analysed by orthogonal projection. Note the comparable RACK1 cytosolic signal on dermal melanoma cells and epidermal keratinocytes. High levels of RACK1 are seen in cutaneous and metastatic melanoma cells with perinuclear localization. Furthermore, in metastases, RACK1 is seen within the nuclei, as indicated by yellow arrowheads on the optical slice and the orthogonal projections. Dotted lines in (A1) and (A2) indicate epidermis-dermis boundaries. e, epidermis; d, dermis. Bar = 5 μm.

In cutaneous melanoma, nests of MITF+ cells expressed RACK1 protein (Figure 4A). The RACK1 signal was scattered in the cytoplasm, mostly in the perinuclear area (Figure 4A). In metastases of melanoma to lymph nodes, lung and heart, RACK1 protein was abundant on MITF+ cells (Figure 4B–D). The RACK1 signal was cytoplasmic in MMC. However, an additional labelling on nuclear punctae was observed in 15% of MMC (Figure 4B–D, yellow arrowheads). Thus, in MeLiM, RACK1 overexpression in tumoral tissues was observed in the cytoplasm of melanoma cells at different stages of progression, from cutaneous melanoma to melanoma metastases, with an additional nuclear localisation in MMC.

### Nuclear RACK1 in pig melanoma cells is not associate with BDNF expression

Nuclear translocation of RACK1 has already been recorded [14] and was shown to mediate the induction of *BDNF *[15]. Since melanoma metastases express BDNF more frequently than primary melanomas [16], we checked whether BDNF would be expressed in MeLiM metastases. No BDNF expression was found in melanoma cells of MeLiM metastases (data not shown).

### High levels of RACK1 in human melanoma

**Figure 5:**
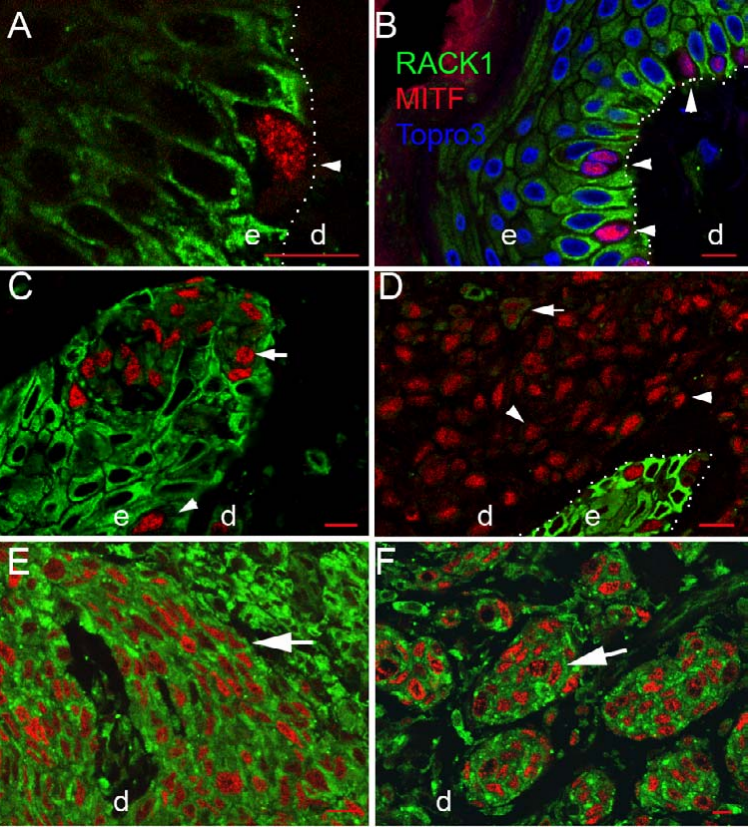
**Cellular distribution of RACK1 in human cutaneous melanocytic proliferations**. Confocal microscopy analysis of double labelling of RACK1 protein (green fluorescence), with MITF (red fluorescence). (A) Control human skin: an MITF-positive melanocyte is localised to the basal membrane. (B-D) Nevi: lentiginous proliferation in B, junctional nest of melanocytes in C, with an additional dermal component in D. (E, F) Cutaneous melanoma samples. Basal and suprabasal keratinocytes display a strong cytoplasmic RACK1 signal. RACK1 is almost not detected in normal melanocytes (A). This also holds true in hyperproliferative lesions of nevi (B-D). In some nevi, RACK1 heterogeneous expression is recognized in melanocytic cells (C). By contrast, in cutaneous melanoma, all MITF+ cells displayed a strong RACK1 signal (big arrow in E and F). Sections of skin are from 6 different patients. Arrowheads point to melanocytes where RACK1 is not detected. Arrows indicate melanocytes expressing cytoplasmic RACK1. Nuclear counterstaining is shown in blue in B. Dotted line indicates epidermis-dermis boundary. e, epidermis; d, dermis. Bar = 10 μm.

To further investigate the expression of RACK1 in human melanoma, we ascertained its presence in a series of samples. Formalin fixed tissue from 4 normal skin biopsies, 14 nevi, 5 cutaneous and 18 metastases of melanoma were analysed using MITF as melanocytic marker [17]. The results are summarized in Table 3. In normal skin, RACK1 was present in negligeable amounts in melanocytes whereas adjacent keratinocytes displayed a strong cytoplasmic signal (Figure 5A). In nevi, RACK1 signal was heterogenous within a given nevus and between nevi showing two patterns: in 7 out of 14 nevi, RACK1 signal was either not detectable (arrowheads, Figure 5B–D) or faint (arrow, Figure 5D). When detected in epidermal or dermal melanocytes, the signal was essentially membranous. In the 7 other nevi, a cytoplasmic or perinuclear signal was observed in nests of epidermal (arrow, Figure 5C) as well as in dermal melanocytes. By contrast, in cutaneous melanoma, whether superficial spreading melanoma or recurrent dermal melanoma, MITF+ fusiform dermal cells as well as dermal nest of melanocytic cells displayed a strong RACK1 cytoplasmic signal which was homogeneous over the whole lesion (Figure 5E, F respectively). In summary, RACK1 signal was low in nevi and very much increased in cutaneous melanoma.

**Table 3:** RACK1 level expression and localisation in normal skin, nevi and melanoma from patients

Tissue type	Age of patient (year-old)	Number of specimens examined	RACK1 expression level		
			
			Undetected or faint membranous	Low cytoplasmic	High cytoplasmic
Normal skin melanocytes		4	4	0	0
Benign nevi	26 to 73	14	7	7	0
Cutaneous melanoma	34 to 79	5	0	0	5
Nodal metastatic melanoma	33 to 80	13	0	0	13
Liver metastatic melanoma	36 to 69	5	0	0	5

**Figure 6:**
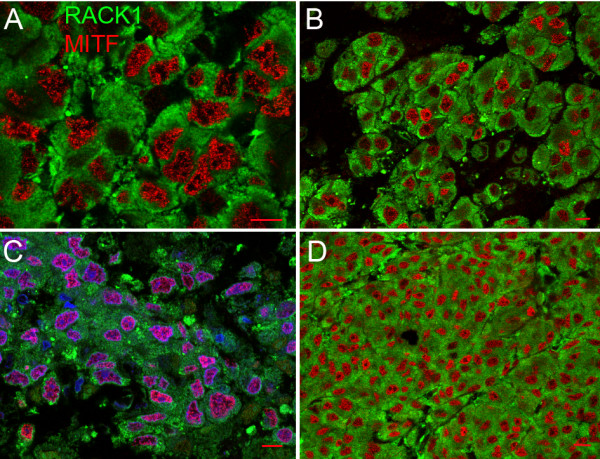
**RACK1 in human melanoma metastasis**. Confocal microscopy analysis of double labelling of RACK1 protein (green fluorescence) and MITF (red fluorescence). (A, B) Melanoma metastasis in lymph node. (C, D) Melanoma metastasis in liver. High levels of RACK1 are seen in the cytoplasm of all metastatic human melanocytes. Sections are from 4 different patients. Nuclear counterstaining is shown in blue in D. Bar = 10 μm.

In lymph nodes as well as in liver metastases, cells presented a strong granular, regular cytoplasmic pattern of RACK1 distribution underlining cell shape (Figure 6A–D). RACK1 overexpression was consistently observed in all MITF+ cells from each of the 23 malignant melanoma samples examined. No nuclear RACK1 labelling was found in these melanoma samples. In summary, overexpression of RACK1 was detected in human melanoma with no apparent changes between cutaneous lesions and metastatic melanoma.

Altogether, screening of human lesions indicates a differential expression of RACK1 in nevi and melanoma. In benign nevi, RACK1 signal was low and heterogeneous on melanocytic cells. By contrast, RACK1 signal presented two distinct features in melanoma: a dramatic increase of intensity and an homogeneous cytoplasmic distribution over the lesion.

### Activated PKCαβ detected in situ in human melanoma cells

**Figure 7:**
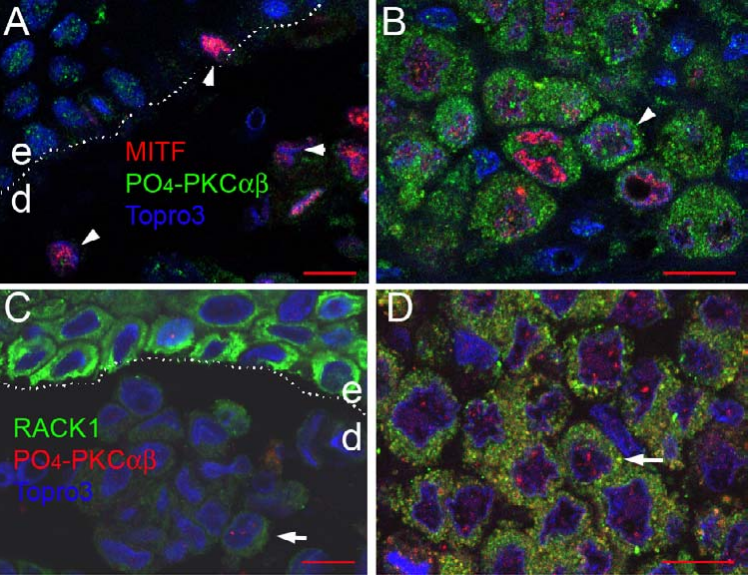
**Cellular distribution of activated PKC in human nevi and melanoma**. (A, B) Confocal microscopy analysis of double labelling of phospho-PKCαβ protein (green fluorescence, biotin amplified signal) and MITF (red fluorescence). (C, D) Confocal microscopy analysis of double labelling of RACK1 protein (green fluorescence, biotin amplified signal), with phospho-PKCαβ (red fluorescence). (A, C) Nevi. (B, D) Melanoma metastasis in lymph node. High levels of activated PKC are seen on MITF-positive melanoma cells in metastasis compared to MITF-positive melanocytes in nevus (arrowheads in B and A, respectively). Dermal melanocytes in nevus display low cytoplasmic RACK1 and nuclear phospho-PKC signals (arrow in C). Abundant signals for RACK1 and activated PKCαβ partially colocalise (arrow in D). Nuclear counterstaining is shown in blue. Dotted line indicates epidermis-dermis boundary. e, epidermis; d, dermis. Bar = 10 μm.

Finally, we checked whether activated protein kinase C (PKC) was a partner of RACK1 in melanoma cells. Double labelling for MITF and phospho-PKCαβ showed a distinct increase in PKC signal in metastases compared to nevi (Figure 7A, B). When double labelling for RACK1 and phospho-PKCαβ was performed, both signals were observed in the cytoplasm of MMC (Figure 7C, D). Both proteins were expressed at higher levels in MMC compared to nevi and there was a partial cytoplasmic colocalisation. An additional nuclear signal for phospho-PKCαβ was detected in epidermal (not shown) and dermal (Figure 7C) melanocytes and in metastatic melanoma cells (arrow in Figure 7D). In cutaneous melanoma, phospho-PKCαβ signal was heterogeneous, lower or at the same level than in MMC (data not shown). These data suggest that PKCα and/or β are involved in the functional role of RACK1 in metastatic melanoma.

Taken together, these results demonstrate that *RACK1 *mRNA and protein are up-regulated in human melanomas as found in pig melanomas, and demonstrate a correlation between the melanoma tumoral status and high levels of RACK1.

## Discussion

Few animal models exist for cutaneous malignant melanomas. The murine models require a combination of gain of function and loss of function mutations in a proto-oncogene and tumor suppressor gene, respectively [18]. Severe sunburn in newborn mice may be needed to induce skin melanomas with high penetrance [19]. By contrast, the MeLiM swine model exhibits spontaneously cutaneous melanomas with histopathological features similar to those of human melanomas [1]. Here, we provide evidence that the hereditary disorder in MeLiM is useful for the identification of regulatory complexes involved in the development of melanomas in humans. Indeed, we found that the adaptor protein RACK1 in pig melanoma is overexpressed as in human melanoma. This is the first time that a prediction based on the MeLiM model finds confirmation in human melanoma. This suggests that similar mechanisms may be operating in the malignant transformation of melanocytes in pigs and man.

Global gene expression profiling on melanoma using various techniques including SAGE has been reported. The nature of the starting material determines the outcome of the comparisons. Noteworthy, the results seem complementary, and differential expression of *RhoC *[20], *WNT5a *[21], *NOTCH2 *[22], *Ubc9 *[23] and genes associated with calcium signalling [24] among others, highlighted pathways involved in cell motility, tissue invasion and resistance to apoptosis. In our SAGE analysis, the *RACK1 *tag was more abundant in the library established from primary culture of metastatic melanoma cells than in the library from normal skin melanocytes. Consistently, *RACK1 *mRNA and protein were not detected in normal epidermal melanocytes, while they were both found at high levels in tumoral cells of cutaneous and metastatic melanomas. RACK1 was barely detected in melanocytes of normal skin in pigs and humans. This is opposed to cultured pig and human melanocytes, in which RACK1 mRNA and protein expression is readily detected [25,26] (and our observations). One explanation to this discrepancy is the microenvironment itself : in culture, melanocytes are grown as a pure population of cells with close interaction [27], in a medium containing factors known to activate the RACK1 promoter [28]. It is noteworthy that melanoma cell lines express even more RACK1 than cultured melanocytes [26]. The latter observation suggests that RACK1 upregulation in MMC was maintained while deriving the corresponding melanoma cell lines. Hence, it is plausible that RACK1 upregulation in melanomas, once established, does not depend on environmental signals provided by stromal cells but is stably inherited from cell to cell.

This study shows that *RACK1 *mRNA and its corresponding protein are systematically overexpressed in tumoral cells of cutaneous and metastatic melanomas. This holds true for all pig and human samples examined to date, whether pigmented or not. Instead, we detected a conspicuously lower expression in nevi. *GNB2L1 *encodes RACK1, an adaptor protein that modulates signaling from PKC, Src, and β-integrin in various systems [29-31]. Evidence for a role of RACK1 in the pathogenesis of melanoma comes from the recent discovery of the capacity of RACK1 to increase survival of MeWo human melanoma cells following UV induced-apoptosis [32]. Moreover, inhibition of RACK1 expression using siRNA was shown to reduce the tumorigenicity of MeWo in a xenograft tumor model [32]. These authors further proposed a role for RACK1, specific to melanoma, involving a crosstalk between ERK and JNK signaling [26]. In this model, RACK1 was shown to induce JNK activation by binding activated PKC, but the PKC isoforms involved were not identified.

PKC is a family of lipid-regulated serine/threonine kinase isozymes which differ in their activation and are differentially expressed in tissues and cell types [33]. Upon activation, PKC translocates subcellularly by specific binding to anchoring proteins like RACK1 [29]. PKC, the main target of TPA, has important roles in cell-cycle regulation, cell survival, malignant transformation and apoptosis [34]. PKC in melanocytic lineage has been widely studied because TPA is essential for *in vitro *growth of normal melanocytes [35,25] and because it was shown to affect the metastatic potential of these cells [36]. Again, studies on cultured melanocytes and melanoma cell lines do not always reflect the actual situation in the whole organism. Hence, the contribution of PKC in melanoma is still controversial. *WNT5A*, identified as a robust marker of highly invasive human melanoma cells [21] was shown to mediate motility through activated classical PKCs [37]. An additional effect of PKC on melanoma was recently underlined by a study of the aberrant expression of claudin-1 in melanoma [38]. Conversely, PKCβ expression was reported lost in 90% of melanoma cell lines [39-41]. Recently, the expression of PKCα and δ isoforms, in contrast to PKCβ, was reported in skin and lung melanoma sections, but it was not specified whether the antibodies recognized the activated isoforms or not [41]. Based on these observations, we analysed phospho-PKCαβ expression in human melanomas. The activated PKCαβ signal was stronger in malignant samples, but only partially colocalised with RACK1. RACK1 is known to bind activated PKCβ II [42] and to a lesser extent activated PKCα [43]. Nuclear signal of phospho-PKCαβ could be ascribed mainly to PKCα [44] although nuclear PKCβ II has also been reported [45]. Altogether, our results on the partial colocalisation of RACK1 and phospho-PKCαβ signal suggest that RACK1 binds more likely to activated PKCβ II than to PKCα in melanoma cells. The remaining RACK1 interacts probably with other PKC isoforms like PKCδ [41,37] or other proteins.

Few studies have detected up-regulation of RACK1 in human cancer specimens [13,46]. *RACK1 *mRNA was found to be strongly expressed in five non-small cell lung carcinomas, mainly in the endothelium of large vessels [13]. *RACK1 *mRNA was also highly expressed in 11 cases of colorectal cancer, with a stronger expression in carcinoma cells than in the stroma [46]. Recently, *RACK1 *upregulation was found to be part of the gene signature associated with shorter metastasis-free survival in breast cancer patients [47]. RACK1 contains seven internal WD40 repeats which confer either stable or reversible binding capability to other proteins. Almost 60 proteins interacting with RACK1 have been described to date. RACK1 could affect cell transformation at multiple levels, increasing proliferation rate, migratory capacity, anchorage independent growth, or resistance to apoptosis [48].

Histopathological features of early stages of melanoma are still ill defined. Clearcut prognostic markers for melanoma which could be used to stratify patients for adjuvant treatments are lacking. Although this study is fairly descriptive, it provides a thorough analysis of RACK1 immunohistochemical detection in human melanoma samples at different stages. The number of samples in our study was limited and should be further increased, but the clear difference in the level and in the homogeneity of RACK1 expression between melanocytic cells in nevi and in cutaneous or metastatic melanoma suggests that RACK1 antigen could be used as a marker of malignancy in human melanocytic proliferation.

## Conclusion

We found that RACK1 overexpression characterized cutaneous melanoma in the MeLiM swine model as well as in human patients. We propose that RACK1 immunolabelling could be used as a potential marker of malignancy in melanocytic proliferation. Our work supports the view that the MeLiM strain provides a relevant model to study the complex mechanisms involved in melanocytic malignant transformation in humans. The additional data issued from our SAGE analysis will probably help in discovering other proteins not yet identified as involved in melanoma pathophysiology and diagnosis of melanoma.

## Methods

### Pig (*Sus scrofa domestica*) tissues

Affected MeLiM males were mated with healthy Duroc or MeLiM sows at the National Institute for Agricultural Research (INRA, Jouy-en-Josas, France) [1,4]. Animal care and use in this study were approved by the INRA ethics committee, in accordance with European Union standards. Biopsies from 3 month-old or younger MeLiM, bred either in France (n= 13) or in the Czech Republic (n = 3), were used. They included cutaneous melanoma consisting of superficial spreading melanoma (n = 2) and nodular melanoma (n = 7) and metastases in lymph nodes (n = 13), lung (n = 10), liver (n = 1), heart (n = 2) and spleen (n = 3), as well as healthy skin (n = 10). Samples of dorsal cervical skin of a healthy pigmented Meishan pig were used as controls. Collected tissues were fixed in 4% buffered paraformaldehyde (PFA) and embedded in paraffin.

### Isolation of metastatic melanoma cells and culture of control melanocytes

Tumor biopsies of lung from a young MeLiM were used to isolate melanoma cells. Conditions for primary cultures of pig melanocytic cells were as described [7]. TPA was added on the second day of culture. After 48 hours cells were rinsed, lysed in Dynabead mRNA direct kit binding buffer (Dynal, Invitrogen, Cergy Pontoise, France) and frozen in liquid nitrogen. Control melanocytes were the non transformed PigMel cells at passage 37 [7].

### Construction of SAGE libraries

Libraries were generated using the SAGE adaptation for downsized extracts (SADE) method using Sau3A as the anchoring enzyme, as described in [49] with a centrifugation of cell lysates to discard melanin. One thousand clones from each library were sequenced. Sequencing reactions were performed by MWG (Martinsried, Germany).

### Tag identification and cloning of the probes

SAGE tags were extracted from sequence files and processed to remove duplicate ditags, linker sequences and repetitive tags using the SAGE 2002 version 4.5 software [6]. Statistical significance was determined using Monte Carlo simulation analysis included in the SAGE software. A *P *value of less than 0.05 was considered significant. Tags were identified using the mammalian Genbank database [50] analysed by the SAGE software, or the [51]. Pig *RACK1 *partial cDNA corresponding to the nucleotide sequence -70 to 900 bp from the ATG start codon was subcloned into pCR4TOPO plasmid (Invitrogen). The resulting plasmid was linearized with *NotI *or *Pme*I to obtain sense or antisense RNA probes, respectively. *In vitro *transcription was performed as described [52].

### *In situ *hybridisation of heavily pigmented samples

*In situ *hybridisation was performed as described [53] with modifications to bleach the sections. Briefly, deparaffinized 5 μm sections were treated for 15 minutes with 0.075% KMnO_4 _and discoloured for 1 minute in 5% oxalic acid with brief rinses between and after treatments. Sections were fixed for 20 minutes in 4% PFA, rinsed, dehydrated and air-dried. Sense or antisense radiolabelled riboprobes at about 15 × 10^6 ^cpm/ml were hybridised as described [52]. Slides were exposed to Biomax MR films (Kodak, France) for 3 days, then dipped in Kodak NTB2 emulsion and exposed for 4 weeks.

### Human tissues

Human melanoma tissues were obtained at the Curie Institute (Paris, France) from patients undergoing lymphadenectomy (n = 13), hepatectomy (n = 5) or epidermal cutaneous resection (n = 19). Cutaneous melanoma specimens consisted of 2 superficial spreading melanoma stages IV (Breslow depth of 2,35 and 2,5 cm) and 3 recurrent dermal cutaneous melanoma. Samples from 37 patients, 25 women and 12 men, were examined. Normal skin from breast plastic surgery was used as control (n= 4). Tissues were fixed either in 4% PFA (n = 38) or in Bouin fixative (n = 2) and embedded in paraffin

### Antibodies (dilutions for immunolabelling)

Mouse monoclonal antibodies were anti-MITF (1:50) (Zymed, Clinisciences, Montrouge, France) and anti-RACK1 (1:150) (Transduction Laboratories, BD Biosciences, Le Pont de Claix, France). Rabbit PEP-8 anti-DCT (1:1000) was obtained from Dr. Hearing and anti-phospho PKCαβ (1:50) was polyclonal (Cell Signaling, Ozyme, France). Cross reaction and specificity of the antibodies to pig tissue were checked by Western blot.

### Immunostaining and confocal microscopy

Antigen retrieval was performed by microwaving deparaffinized sections in citrate buffer, pH 6, for pig sections, or in Tris-EDTA, pH 9, for human sections. For immunohistochemistry, primary antibodies were reacted with the avidin-biotin complex (ABC Elite, Vector, Biovalley, France). For double immunofluorescence, antibodies applied overnight at 4°C were revealed with anti-mouse isotype or anti-rabbit antibodies, one labelled with Alexa Fluor 555, the other coupled to biotin and revealed with Alexa Fluor 488-labelled streptavidin (Molecular Probes, Invitrogen, France). Nuclear counterstaining was achieved with Topro 3 (Molecular probes). Sections were observed with a Leica laser TCS SP2 scanning confocal microscope producing 0.7 μm-thick optical sections. Controls without the first antibodies showed no unspecific labelling. Confocal images were processed with the computer program Leica Lite or Zeiss LSM Image Browser for orthogonal projections. All images shown are individual sections of z series, plus the orthogonal projections of the stack when indicated. Final Figures were assembled with Adobe Photoshop (Adobe Systems, USA).

## Competing interests

The authors declare that they have no competing interest.

## Authors' contributions

GE carried out SAGE libraries from mRNA to TAG identification, *in situ *hybridisation, immunofluorescence confocal studies and drafted the manuscript. SJ carried out cell culture, and participated to the rest of the work. PB contributed to the cell culture and the library construction. FB participated to the pathological characterization of pig samples and in the drafting of the manuscript. CG, SV–N and VH contributed to pig breeding and sample collection. XS–G supplied human samples and participated in their pathological characterization. J–JP together with GE, SJ, and PB conceived the study, participated in its design and coordination and carried out the final drafting to the manuscript. All authors read and approved the final manuscript.
